# Ultramorphological Characteristics of *Falsogastrallus sauteri* Pic (Coleoptera: Ptinidae) and a New Species of *Cephalonomia* Westwood (Hymenoptera: Bethylidae): A Book-Boring Beetle and Its Natural Enemy in Taiwan

**DOI:** 10.3390/insects11040223

**Published:** 2020-04-03

**Authors:** Yu-Hsiang Ho, Yun Hsiao, Mamoru Terayama, Mei-Ling Chan

**Affiliations:** 1 Department of Entomology, National Chung Hsing University, Taichung 40227, Taiwan; b123b44@smail.nchu.edu.tw; 2 Australian National Insect Collection, CSIRO National Research Collections Australia, Canberra, ACT 2601, Australia; yunhsiao@outlook.com; 3 Division of Ecology and Evolution, Research School of Biology, The Australian National University, Canberra, ACT 2601, Australia; 4 Department of Agriculture and Agricultural Life Sciences, The University of Tokyo, Tokyo 113-8657, Japan; terayama@fa2.so-net.ne.jp; 5 Division of Biology, National Museum of Natural Science, Taichung 40453, Taiwan

**Keywords:** bookworms, *Falsogastrallus sauteri*, parasitoid wasps, *Cephalonomia* wasps, old books, historic library, morphology, new species, Taiwan, Anobiinae

## Abstract

Libraries are invaluable resources, documenting significant events that have shaped human history. However, the preservation of old books is severely threatened by insects commonly referred to as bookworms. In this study, a sample of infested books in a historic library in Taiwan was randomly selected and examined. An anobiid book-boring beetle, *Falsogastrallus sauteri* Pic, 1914 (Coleoptera: Ptinidae) was identified as the major bookworm species present. To facilitate its identification, both adults and larvae of *F. sauteri* are redescribed, with emphasis on its ultramorphological characteristics as revealed by scanning electronic microscopy. Furthermore, an undescribed parasitoid wasp in the Bethylidae was discovered in the frass, holes and tunnels created by *F. sauteri.* The new species, *Cephalonomia* *formosiensis* sp. nov. is described, and we suggest that it probably uses *F. sauteri* as host.

## 1. Introduction

Books with cellulose and additives such as gelatin, starch and animal glue can provide nutrition for insects [1]. Many insects can damage books and cause major problems for the historical archival management in libraries. The insect groups that cause the majority of damage are the beetles (Coleoptera), silverfish (Zygentoma), termites (Blattodea) and moths (Lepidoptera). Among them, the larvae of certain beetles are most frequently termed bookworms, and they can cause serious damage to books. Anobiids (Formerly Anobiidae) are the largest group of book-eating beetles. They were first recognized as Anobiidae by Pic [2]. Recently, Borowski and Zahradníc [3] replaced the family name Anobiidae with Ptinidae because of priority, and Bell and Philips [4] confirmed their monophyly based on the molecular evidence. Many anobiid species, e.g., belonging to the genera *Anobium*, *Ernobius*, *Gastrallus*, *Falsogastrallus*, *Hadrobregmus*, *Lasioderma*, *Nicobium*, *Oligomerus*, *Stegobium*, *Priobium*, *Ptilinus*, *Sculptotheca*, *Tricorynus*, *Trichodesma* and *Xestobium*, have been reported as pests of cultural heritage items [5,6,7,8,9]. Species that have been shown to infest papers and books, which are commonly referred to as bookworms, include *Lasioderma serricorne* (Fabricius), *Anobium punctatum* De Geer, *Stegobium paniceum* (Linnaeus), *Falsogastrallus sauteri* Pic, *Tricorynus herbarius* (Gorham), *Nicobium castaneum* (Olivier) and *Xestobium rufovillosum* (De Geer), which can infest papers or books [7,10,11,12].

A diversity of factors can influence anobiid preferences for certain cellulosic or additive materials. For example, books with higher starch and sugar levels may be more attractive, as cellulose alone is not enough for larval development [7,10,12]. White [12] reported *T. herbarius* as a pest of historical books in United States and found some type of papers, such as clay-filled and chemically treated papers, are less attractive; damp books infested by brown mold were also immune to damage. Humidity is another important factor influencing susceptibility to beetle damage, as damp environments can increase book deterioration and ultimately facilitate insect attack.

In previous studies in Taiwan, three species of anobiid beetles, *L. serricorne*, *S. paniceum* and *F. sauteri*, were found damaging books in libraries [13,14,15,16], but there has been no further survey or detailed information so far. In 2017, we received a batch of books from a historic library in central Taiwan accidentally. From those damaged books published from 1862 to 1974, we collected numerous beetles and parasitoid wasps. The beetles were identified as Sauter anobiid, *F. sauteri*, and the wasps are here described as a new species of Bethylidae in the genus *Cephalonomia* Westwood.

The genus *Falsogastrallus* belongs to the tribe Gastrallini. It comprises 16 species, with 6 species described from the Palearctic region, 4 from the Oriental, 1 from the Nearctic and 2 from the Neotropical region [16]. There is only one species, *F. sauteri*, recorded in Taiwan. Sakai [14] redescribed *F. sauteri*, and Xiong et al. [17] observed the morphology of the adults and larvae using scanning electron microscopy (SEM). However, few ultramorphological characteristics of *F. sauteri* were mentioned.

The family Bethylidae, with 2920 described species in 96 genera [18], are a group of wasps, usually 1 to 20 mm in length, which are parasitoids of Lepidoptera and Coleoptera larvae [19]. The genus *Cephalonomia* comprises 44 species and is distributed all over the world except the Antarctic region [20,21,22]. In East Asia, nine species have been known up to present [5,22,23,24,25], and two species have been found in Taiwan [26]. Polymorphism is known in several species in this genus [19,27]. In *C. gallicola* (Ashmead) and *C. urichi* Brues, the adult males are either apterous or macropterous (fully winged) [27,28,29]. In *C. formiciformis* Westwood, the adult females are either macropterous or brachypterous [27,30], and in *C. perpusilla* Evans, the adult females are either macropterous, micropterous, subapterous or apterous, and the adult males are macropterous or apterous [31,32].

In this study, we examined all the damaged books and interpreted our observations of damage to the books. *F. sauteri* is redescribed based on specimens from Taiwan, the type locality of this species. Its ultramorphological characteristics were also investigated and are illustrated. Furthermore, we describe the new species of *Cephalonomia* and discuss its ecological relationship to *F. sauteri*.

## 2. Materials and Methods

### 2.1. Specimen Collecting

We investigated the old books preserved in a library in central Taiwan. Forty-six books were randomly chosen from a number of infested books either on the open library shelves or in the storage room following by librarian’s instruction, and the damaged books were carefully checked. The frass were shaken from those books and carefully examined to collect hidden living organisms or corpses. Tunnels bored by *F. sauteri* in the books were examined under a microscope and observations were recorded. The window frames were also searched to find any corpses of insects. Most specimens of *F. sauteri* and *Cephalonomia* sp. were collected from the old, damaged books, and some of the *F. sauteri* specimens were collected from the window frames in the library.

### 2.2. Specimen Examination and Deposition

Adult specimens of *F. sauteri* for morphological studies were preserved dry, and *Cephalonomia* specimens were directly preserved in 70% ethanol. Immature specimens were preserved in 75% ethanol. Adult specimens of *F. sauteri* used for SEM photographs were dehydrated in a series of ethanol baths and were then put in an oven under 45 °C to be dried overnight. Subsequently, the specimens were fixed on the stub and then coated with Pt–Pb alloy using ion-beam sputter coater. Larval specimens used for SEM photographs were firstly fixed by 2.5% glutaraldehyde in 0.2 M cacodylate buffer and dehydrated by means of a series of ethanol baths (50%, 60%, 70%, 80%, 90%, 95% and 100%). After the process of critical point drying (CPD), the specimens were fixed on the stub and then coated as above. Photographs were taken with the Hitachi SU1510 scanning electron microscope (VP-SEM). 

Dissection of male genitalia of *F. sauteri* were performed as follows. The abdomen was removed, soaked in high-concentration KOH solution for approximately one hour and then cleaned in distilled water for a while. The abdominal sternites were removed and aedeagus drawn out for further examination under a stereomicroscope. Colored photographs were taken with a Canon EOS 650D camera, attached to a Leica S8 APO microscope and subsequently stacked with Helicon Focus 5.3 Pro.

Two paratypes of the new species of *Cephalonomia* will be deposited in the Taiwan Agriculture Research Institute (TARI), Taichung, Taiwan and two in the University of Tokyo (TU), Tokyo, Japan; all other specimens, including the holotype, will be deposited in the National Museum of National Science (NMNS), Taichung, Taiwan. The nomenclatural acts established herein are registered under ZooBank LSID urn:lsid:zoobank.org:pub:6E39671D-81FB-42B0-B891-3283F4143E3F.

### 2.3. Terminology

The terminology used in the description of *F. sauteri* follows Sakai [14] and Philips and Bell [33], and that of *Cephalonomia* species follows Terayama [26]. 

Abbreviations used for the measurements of *F. sauteri* are as follows:

Total length (TL): total length of outstretched individual, from the clypeus to the abdominal apex.
Total width (TW): maximum width of outstretched individual.

Abbreviations used for the measurements of *Cephalonomia* are as follows:

Head length (HL): maximum length of head excluding mandibles in full-face view.
Head width (HW): maximum width of head including eyes in full-face view.
Width of frons (WF): minimum width between eyes in full-face view.
Eye length (EL): maximum length or height of eye in lateral view.
Diameter of anterior ocellus (DAO): maximum diameter of anterior ocellus.
Width of ocellar triangle (WOT): distance across and including posterior ocelli.
Posterior ocellar-line (POL): shortest distance between posterior ocelli.
Antero-posterior ocellar-line (AOL): shortest distance between anterior ocellus and posterior ocellus.
Ocello-ocular line (OOL): shortest distance from a posterior ocellus to nearest eye margin.
Length of mesosoma (LM): maximum diagonal length of mesosoma excluding the pronotal collar, in lateral view.
Length of propodeal disc (LPD): measured along the midline excluding posterior declivity, in dorsal view.
Width of propodeal disc (WPD): maximum width of the disc excluding the anterior potion from the level of propodeal spiracles, in dorsal view.
Forewing length (FWL): maximum length of forewing.
Total length (TL): total length of outstretched individual, from the mandibular apex to the metasomal apex.

## 3. Results

### 3.1. Examination of Books Damaged by Falsogastrallus sauteri and the Frass from the Books

**Figure 1:**
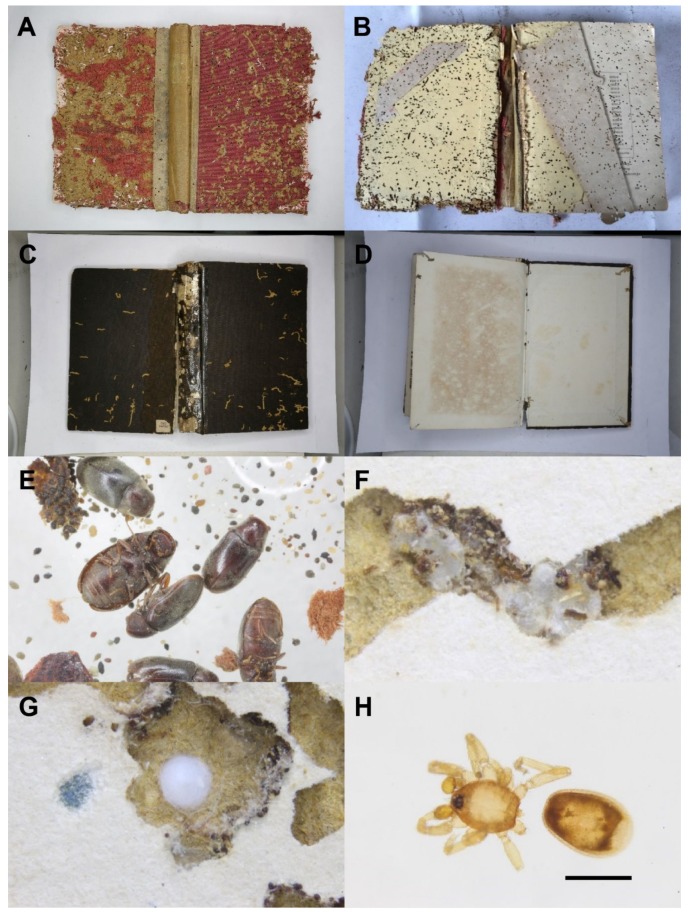
Damaged books and associated arthropods. (**A**) The front hardcover of a damaged book published in the UK and (**B**) the inside front hardcover. (**C**) The front hardcover of a damaged book published in Japan and (**D**) the inside front hardcover. (**E**) Corpse and frass of *Falsogastrallus sauteri*. (**F**) Silk cocoons of *Cephalonomia* wasps in a tunnel. (**G**) Egg sac of an oonopid spider. (**H**) Oonopid spider found in a tunnel (scale = 0.5 mm).

The 46 infested books we received from the library were recorded and checked (Figure 1A–D). The years and countries of publication are shown in Table 1. These books are mostly dated from 1862 to 1943 while one book was published in 1974. All books were hardcovered, with only three books paperbacked. In terms of the publishing countries, 44% of books were published in the UK (Figure 1A,B), and 22% of books were published in Japan. Most books from the UK were characterized by the degree of damage inside the hardcovers being higher or almost the same as that outside. However, with the books published in Japan (Figure 1C,D), we commonly observed surface tunnels on the outside cover but much less damage on the inside covers. Three books, published in 1901 in the USA, 1910 in Germany and 1974 in The Netherlands, had damage only on the front cover and no damage on either the inside front cover or either side of the back cover. It is likely that the damage observed here was the result of these books being directly adjacent to a book with substantial insect activity. One paperbacked book, published in 1943 in Japan with 20 pages, had neither silk cocoons nor frass and was probably affected by its “delicious” neighbors as well. In some books, water stains seemed to encourage infestation by *F*. *sauteri*.

**Figure 2:**
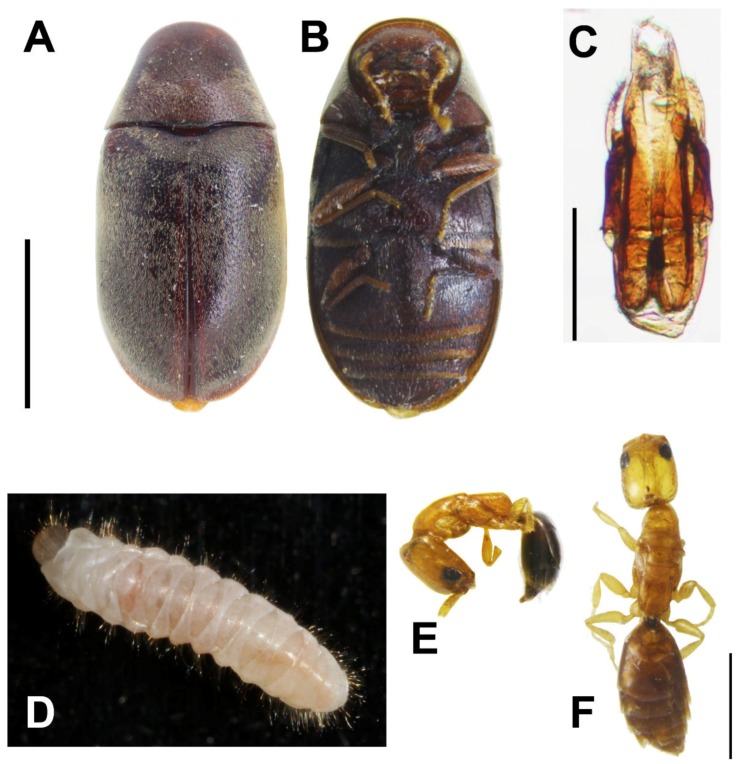
*Falsogastallus sauteri*. (**A**) Adult habitus, dorsal view and (**B**) ventral view (scale = 1 mm). (**C**) Male genitalia (scale = 0.2 mm). (**D**) Larva habitus, dorsal view. (**E**,**F**) *Cephalonomia formosiensis* sp. nov. (**E**) Habitus of brachypterous adult female, lateral view and (**F**) dorsal view (scale = 0.2 mm).

Ignoring dead arthropod fragments, there were more than 200 *F*. *sauteri* dead adults (Figure 1E) and 4 living larvae, more than 40 *Cephalonomia* specimens, 2 oonopid spiders (Araneae: Oonopidae) and a few mites and booklice picked out from the frass. Both *F. sauteri* and *Cephalonomia* wasps were found in higher densities. Additionally, plenty of empty cocoons (Figure 1F) spun by *Cephalonomia* larvae occurred in the frass, and the tunnels suggest that this species was correlated with *F. sauteri*. Interestingly, at least 10 egg sacs (Figure 1G) and 2 individuals (Figure 1H) of oonopid spiders were found in the tunnels inside hardcovers made by *F. sauteri*, suggesting that oonopid spiders used the tunnels as shelters and for food.

The holes on the cardboard of front covers were usually blocked with firmly glued frass by the larvae of *F. sauteri*. When opened, lots of loose and scattered frass was present, and numerous solid and hard frass “walls” built by *F. sauteri* larvae were observed inside the tunnels, which might be the cells built for pupation. The silk cocoons of *Cephalonomia* wasps were mainly found within the tunnels, either forming a string or a cluster. Sometimes, the surface of a cocoon was fully covered by excrement. The egg sacs of the oonopid spiders were about 0.1–0.2 mm in diameter; they were larger, more whitish and stickier than the silk cocoons of *Cephalonomia* wasps.

The cardboard of the book hardcovers were usually the “favorite” parts for *F. sauteri*, especially the area around the edges. Almost all the dead arthropods, silk cocoons of wasps and egg sacs of spiders were found in the cardboard, no matter which side of the cover it was. Generally, the number of holes and tunnels excavated by the larvae gradually decreased passing from the covers into the pages, unless some glued pages or pages with different materials preferred by *F. sauteri* larvae were present.

### 3.2. Taxonomy

#### 3.2.1. *Falsogastrallus sauteri* Pic, 1914

**Figure 3:**
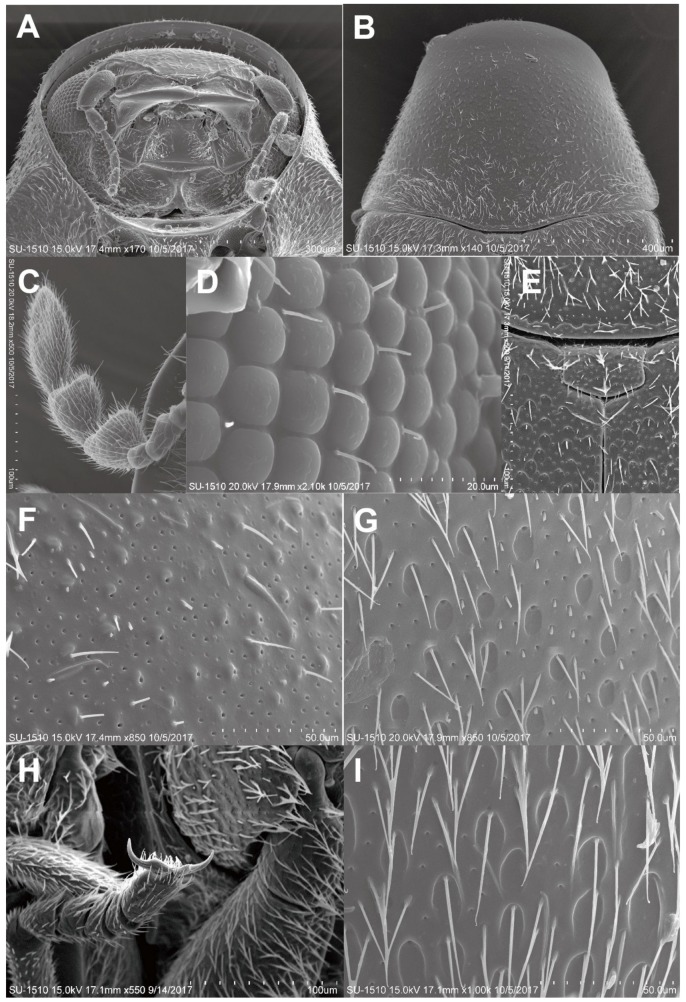
Adult of *Falsogastrallus sauteri*. (**A**) Head, frontal view. (**B**) Pronotum, dorsal view. (**C**) Antenna, dorsal view. (**D**) Compound eye. (**E**) Scutellum, dorsal view. (**F**) Surface of Pronotum, dorsal view. (**G**) Surface of elytron, dorsal view. (**H**) Tarsi and claw. (**I**) Sternite, ventral view.

**Figure 4:**
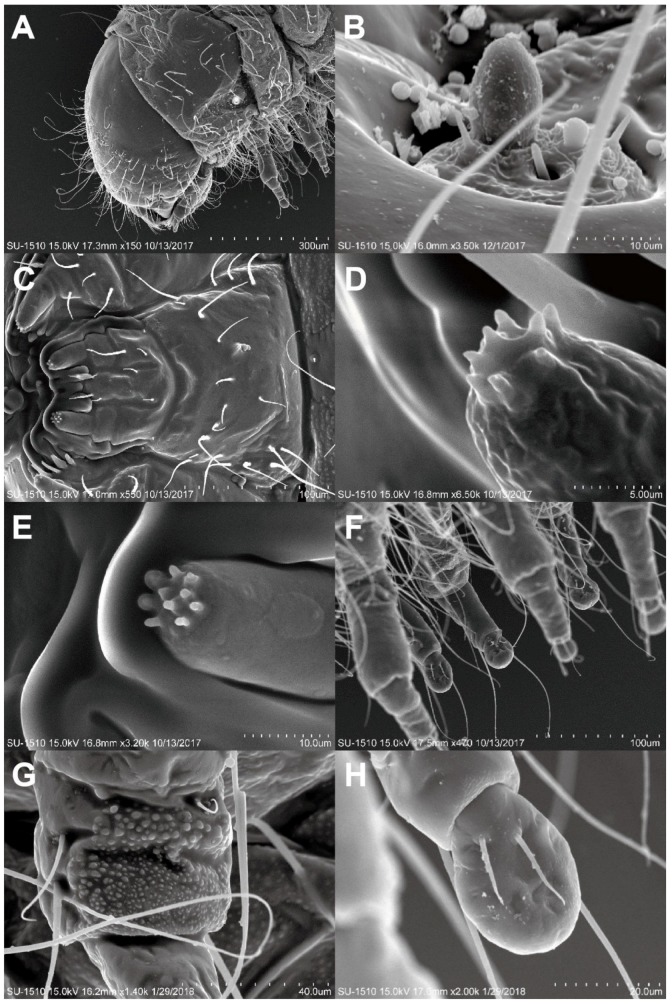
Last instar Larva of *Falsogastrallus sauteri*. (**A**) Head and thorax, lateral view. (**B**) Antennae. (**C**) Mouth part, ventral view. (**D**) Sensilla of maxillary palp. (**E**) Sensilla of labial palp. (**F**) Legs, lateral view. (**G**) Coxa, inner side. (**H**) Tarsungulus, inner side.

(Figure 1E, Figure 2A–C, Figure 3A–I, Figure 4A–H)

*Falsogastrallus sauteri* Pic, 1914: 10. Type locality: Taiwan: Anping, Tainan City.

*Neogastrallus librinocens* Fisher, 1938: 44.

**Material examined.** Fifteen exs., TAIWAN: Taichung, Wufeng, in a library, 17.VII.2017. legs. S. R. Lin and C. H. Liou.

**Diagnosis.** Length range from 1.5 to 3.5 mm, body shape oval, covered by hairs. Head subcircular, covered by the pronotum. Eyes small, widely separated. Antennae 9-segmented. Pronotum strongly convex above. Elytra long and oval-shaped. 

**Redescription** (after Pic, 1914; Sakai, 2003; Philips and Bell, 2010) [14,33,34]. **Adults.** Body (Figure 2A,B). Oblong oval, convex, with length two times width; surface covered with short, dense, appressed, grayish pubescence.

Head (Figure 2B, Figure 3A). Subcircular, deeply sunk within the prothorax, transversally slightly convex; surface finely, densely punctate. Eyes small, slightly globular, ratio of an eye diameter to interocular space 1.0:1.6; facets round and smooth, with few hairs between facets (Figure 3D). Antennae (Figure 3C) 9-segmented; antennomere I elongate oval; II elongate, rounded apically; III–V narrow, slightly triangular, subequal in width; VI extremely small, distinctly narrower than V; VII–VIII triangularly projected inward; IX oblong; ratio of the lengths of antennomeres from base to apex as follows: 1.5:1.0:0.75:0.7:0.75:0.45:1.5:1.5:1.75.

Thorax (Figure 2A, Figure 3B). Pronotum bell-shaped, strongly convex transversely; lateral sides nearly straight, slightly arcuate; surface (Figure 3F) densely, separately punctate. Scutellum (Figure 3E) transversally rectangular. Metaventrite somewhat ocellate–punctate.

Elytra (Figure 2A). Strongly convex, with length two times width, as wide as pronotum at humeri; lateral sides subparallel in basal two-thirds, then gradually narrowed apically; elytral apices rounded; surface (Figure 3G) doubly punctate, the first oval, coarsely, sparsely punctate, larger than the second, puncture diameter the same as distance between punctures, the second minute, finely, densely punctate; each elytron with distinct groove along lateral margin, indistinctly developed near elytral apex. 

Legs (Figure 3H). Slender; pro- and midcoxae widely separated; protibiae unarmed externally; tarsal formula 5–5–5, with length 0.75 times tibial length.

Abdomen (Figure 2B) with four ventrites; abdominal sternite I fused with II, forming ventrite I, sternite I longer than II in middle while short than it at sides; ratio of the lengths of ventrites as follows: 6.9:1.6:1:2; surface (Figure 3I) finely, densely punctate. 

Aedeagus (Figure 2C). Ventral process of each paramere bent inwards, with varying bending degrees among different specimens, apices with two obtuse, rounded tooth-like projections; paramere with palpi-like lobe at lateral sides, expanded and bent inwards apically, with varying bending degrees among different specimens, surface densely covered with hair-like setae; dorsal plate roundly concave in middle; median lobe thick, extending from tegmen in natural condition, with slender, elongate sclerite roundly expanded apically, exceeding ventral process. 

Color. Completely reddish brown to dark brown, with antennae and legs paler.

Measurements (mm). TL 2.08–2.28, TW 1.07–1.14.

**Larva (last instar)**

Body (Figure 2D). C-shaped, elongate, subcylindrical, moderately curved; surface with integument densely papillate, fairly evenly covered with long setae. 

Head (Figure 4A). Rounded, hypognathous; epicranial stem distinct, frontal arms absent. Stemmata apparently absent. Antennae (Figure 4B) vestigial, with a distinct stocky basiconicum sensillum. Frontoclypeal suture absent; clypeolabral region narrow. Mandibles symmetrical, short, stout. Ventral mouthparts (Figure 4C) retracted. Maxilla with distinct transverse cardo, maxillary palps two-segmented, apex with eight distinct columnar sensilla (Figure 4D); labium completely fused to the head-capsule; labial palps two-segmented, apex with 10 columnar sensilla (Figure 4E). Ventral epicranial ridges absent; gular region absent. 

Thorax (Figure 4A). Thorax without any asperity. Prothorax without sclerotizations, large subrectangle smooth area present lateromedially; spiracles located on the prothorax, annular, situated posterolaterally. Mesotergum and metagum each with two transverse plicae.

Legs (Figure 4F). Five-segmented, well developed, nearly cylindrical, different sized papillae forming rough areas occurring on outer side of coxa and inner side posteriorly (Figure 4G), tibiotarsus with pair of long hairs near base outside; tarsungulus (Figure 4H) with bladder-like arolium, two-juxtaposed short setae inside, claw invisible. 

Abdomen (Figure 2D). Ten-segmented, with segment IX well developed, subangled posteriorly, without urogomphi and median process; abdominal segments without any asperity, terga I–VIII usually with two transverse plicae; segment X terminal, below anus, with two oval pads separated by longitudinal groove. Spiracles annular, located on abdominal segments I–VIII.

Color. Creamy white.

Measurements (mm). TL 0.32, TW 0.05.

**Remarks.** The adults of this species can be distinguished from congeners by the combination of the following aedeagal characters: ventral process of each paramere provided with bitoothed apex, which bent inwards; paramere provided with palpi-like lobe at lateral sides, which expanded and bent inwards apically.

**Biology.** This species is a major pest of old books and paper archives, Xiong [35] stated it was the most destructive pest of books and paper archives in China, and many studies of it have been conducted in China [17,35,36]. The life history of *F. sauteri* has also been reported in Chongqing. Under laboratory conditions, the optimum temperature range for development is 18–30 °C, and the life history can be up to a year based on the study of Xiong et al. [36]. The main larval stage is from May to November, and larvae begin overwintering from November to April of the following year, then most adults are active from April to July [36]. The growth rate is slower when the temperature is lower [35,36]. Most damage is caused by the larvae, which eat papery products and sometimes even break the books into fragments. The pupae build their cells between the pages, which makes the book difficult to open. The adults make exit holes on the surface of the books or archives and do not feed anymore [17]. We collected living larvae from the frass on August 2017 and found adults from the same frass on May 2018, which indicates that the life cycle of *F. sauteri* in Taiwan may be similar to that of populations in China. However, a further investigation is required.

**Distribution.***F. sauteri* is mainly distributed in the Oriental region, but it has been introduced to some other countries, such as the USA [14]. It was first recorded in Taiwan from Anping and was described as a new species [34].

#### 3.2.2. *Cephalonomia formosiensis* Terayama & Ho sp. nov.

**Figure 5:**
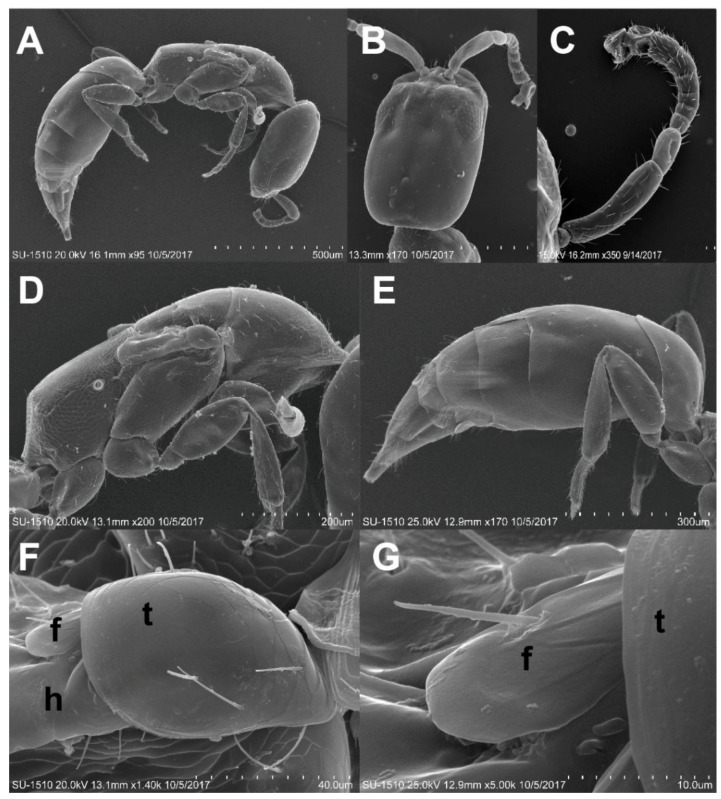
Brachypterous adult female of *Cephalonomia formosiensis* sp. nov. (**A**) Habitus, lateral view. (**B**) Head, dorsal view. (**C**) Antenna. (**D**) Mesosoma, lateral view. (**E**) Metasoma, lateral view. (**F**) Tegula, lateral view. (**G**) Wing, lateral view (t = tegula, f = forewing, h = hindwing).

**Figure 6:**
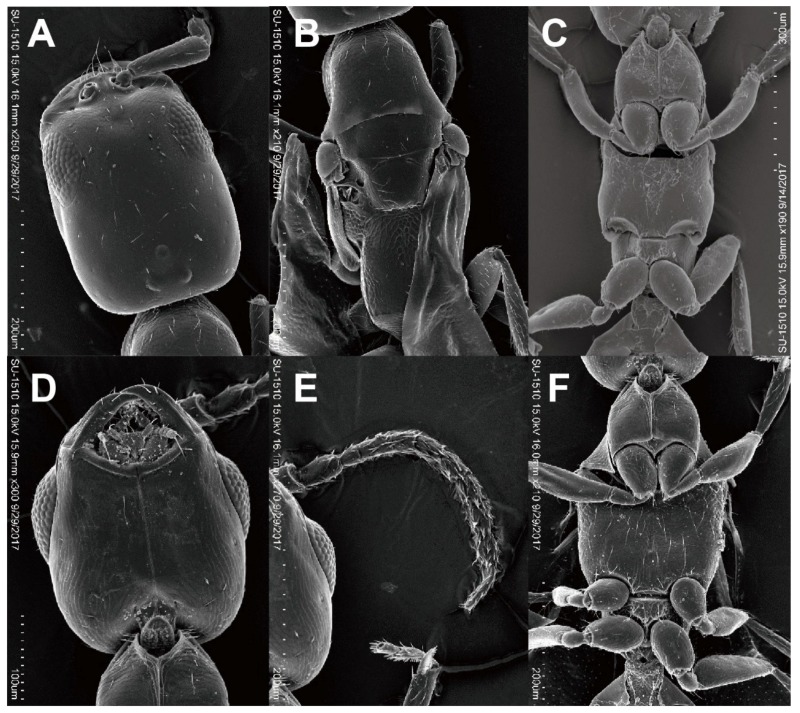
*Cephalonomia formosiensis* sp. nov. (**A–C**) Macropterous adult female. (**A**) Head, dorsal view. (**B**) Mesosoma, dorsal view and (**C**) ventral view. (**D–F**) Adult Male. (**D**) Head, ventral view. (**E**) Antenna, dorsal view. (**F**) Mesosoma, ventral view.

(Figure 2E,F, Figure 5A–G, Figure 6A–G)

**Material examined**.

Type series. Holotype: Brachypterous adult female, TAIWAN: Taichung, Wufeng, in Library of Taiwan Agricultural Research Institute, 17.VII.2017, legs. S. R. Lin and C. H. Liou. Paratype: 11 brachypterous adult females, two macropterous adult females, one male, same data as holotype. 

Non-type material examined. Two brachypterous adult females, one macropterous adult female, one adult male, same data as holotype (specimens for observation with SEM).

**Diagnosis.** Length range from 0.7 to 2.5 mm, with wings fully developed, short, or absent in either sex. Maxillary palpi with four or five segments, labial with one or two segments; clypeus short, its median lobe absent or poorly developed; antenna with 9, 10 or 12 segments; ocelli present (with a few exceptions in the wingless form); mesoscutum without notauli; parapsidal furrows present as narrow lines in the winged form; wing veins simple, radial vein absent, median vein (M + Cu vein) often absent, basal and anal veins both absent; prostigma present. 

**Description. Holotype. Brachypterous adult female.** Structure: Head rectangular, 1.45 times as long as wide, with parallel sides and almost straight posterior margin in full-face view; posterolateral corner round, not forming angle. Maxillary palpi with one segment, labial with four segments. Mandible subtriangular, with two acute triangular teeth; surface smooth, without punctures. Clypeus short, with weakly convex anterior margin. Antenna with 12 segments; first to fifth antennomeres in a ratio of 7:2:1:0.7:0.9 in length; scape 3.9 times as long as wide; pedicel 1.3 times as long as wide; third and fourth antennomeres each shorter than long, sixth antennomere as long as wide. Eye relatively large and even convex, with 13 ommatidia in the longest rows. WF 1.32 times EL. Frons and vertex smooth and shining, without punctures. In lateral view, head 1.89 times as long as high, with gently convex dorsal margin; anterior 2/3 of ventral margin almost straight, posterior 1/3 convex. Ocelli forming acute triangle; POL:AOL = 1.5:2; OOL 3.5 times WOT; frontal ocellus ca. 0.02 mm in diameter. 

Mesosoma slender, 1.40 times as wide as WPD. Pronotal disc wider than long, 0.71 times as long as wide, with convex anterior margin and straight posterior margin in dorsal view; anterolateral corner not forming angle; surface smooth and shining; lateral surface weakly microreticulate. Mesoscutum smooth and shining, with weak and shallow parapsidal furrows; maximum width of dorsal disc 0.86 times WPD; notauli absent; scutellum smooth and shining, slightly longer than mesoscutum; scutellar pits rectangular, separated by their own lengths. Metanotum reduced medially, scutellum almost in contact with propodeum; surface smooth and shining, scattered with a few shallow punctures. Mesopleuron smooth and shining; upper portion with a longitudinal fovea. Propodeum microreticulate including declivity surface and lateral surfaces. Dorsal disc long, rectangular, 1.12 times as long as wide, with subparallel sides and weakly concave posterior margin; lateral carinae present, but transverse carina absent.

Metasoma long and slender, almost as long as mesosoma, surface largely smooth and shining; second tergum 0.30 mm in maximum width in dorsal view. 

Wings short; forewing minute; hindwing oval, 1.25 times as long as wide, slightly exceeding the anterior margin of propodeum. 

Pubescence. Dorsa of head, mesosoma excepting propodeum and metasoma scattered with short suberect to subdecumbent setae; fourth to sixth terga of metasoma with suberect setae moderately; tarsi and antennal funicles much abundant decumbent setae. Eye glabrous (under the stereomicroscope at magnification 160, but several very short hairs recognized in SEM by other specimen).

Color. Head and mesosoma reddish brown; metasoma black; mandible, clypeus, antenna and legs yellow.

Measurements (mm). Brachypterous adult female. Holotype: HL 0.40, HW 0.24, WF 0.17, EL 0.13, LM 0.53, LPD 0.20, WPD 0.18, TL 1.5. Paratype: Measurements (mm; n = 5). HL 0.39–0.43, HW 0.24–0.29, WF 0.17–0.18, EL 0.11–0.13, LM 0.51–0.60, LPD 0.19–0.22, WPD 0.17–0.19, TL 1.4–1.6.

**Macropterous (fully winged) adult female.** General shape of body as in micropterous adult female. Wing membrane hyaline; FWL 0.95 mm; median vein present; pterostigma smaller than prostigma; pterostigma present at basal 1/4 of wing. Color as in micropterous adult female; prostigma and pterostigma brown: wing veins yellowish brown.

Measurements (mm; n = 1). HL 0.40, HW 0.29, WF 0.17, EL 0.11, LM 0.60, LPD 0.20, WPD 0.18, TL 1.6.

**Male.** Structure: Head shorter than that of female (6D), almost as long as wide including eyes, 1.26 times as long as wide excluding eyes, convex posterior margin in full-face view; posterolateral corner round, not forming angle. Maxillary palpi with one segment, labial with four segments. Mandible with two acute teeth. Clypeus short, with weakly convex anterior margin. Antenna with 12 segments, slender than that of female (Figure 6E); first to fifth antennomeres in a ratio of 8:3:1:1:1 in length; scape 4.0 times as long as wide; pedicel 1.8 times as long as wide; third to sixth antennomeres each as long as wide, seventh and eighth antennomeres slightly longer than wide. Eye large and convex (Figure 6D), with about 20 ommatidia in the longest rows. WF 1.36 times EL. Frons and vertex smooth and shining, without punctures. In lateral view, head 1.39 times as long as high, with convex dorsal margin; anterior 2/3 of ventral margin almost straight, posterior 1/3 convex. Ocelli larger than those of female, forming obtuse triangle; POL:AOL = 2:1.2; OOL 1.3 times WOT; frontal ocellus ca. 0.03 mm in diameter. 

Mesosoma wider than that of female (Figure 6F), 1.83 times as wide as WPD. Pronotal disc trapezoidal, wider than long, 0.34 times as long as wide, with slightly convex anterior margin and straight posterior margin in dorsal view; anterolateral corner forming dull angle; surface smooth and shining. Mesoscutum smooth and shining; dorsal disc as wide as WPD; notauli absent; parapsidal furrows shallow and weak; scutellum smooth and shining, as long as mesoscutum; scutellar pits rectangular, separated by separated by 0.5 times their own lengths. Metanotum distinct, 0.3 times as long as mesonotum, scutellum and propodeum separated medially; surface smooth and shining. Mesopleuron smooth and shining; upper portion with a longitudinal fovea. Propodeum microreticulate including declivity surface and lateral surfaces; dorsal disc rectangular, as long as wide, with parallel sides and weakly concave posterior margin; lateral carinae present, but transverse carina absent.

Metasoma long and slender, 2.1 times as long as wide, and almost as long as mesosoma; surface largely smooth and shining; 2nd tergum 0.25 mm in maximum width in dorsal view. 

Wings hyaline; FWL 0.75 mm; median vein present; pterostigma smaller than prostigma; pterostigma present at basal 1/4 of wing. 

Pilosity. Dorsa of head, mesosoma excepting propodeum and metasoma scattered with short suberect setae: third to sixth terga of metasoma each with a row of decumbent setae at near posterior margin; tarsi and antennal funicles much abundant decumbent setae.

Color. Head and mesosoma dark brown; metasoma black; mandible, clypeus, antenna and legs yellow; prostigma and pterostigma brown; wing veins yellowish.

Measurements (mm; n = 1). HL 0.29, HW 0.28, WF 0.15, EL 0.12, LM 0.55, LPD 0.18, WPD 0.18, TL 1.4.

**Remarks.** Among the East Asian congeners, the adult females of *C. gallicola* are always apterous, and *C. fugen* from Japan are micropterous, the remaining species are fully winged. However, the adult females of *C. formosiensis* are either brachypterous or macropterous, and the adult males macropterous. This species is most similar to *C. formiciformis* from Europe, in the dimorphic adult females (macropterous and brachypterous forms). However, it is easily separated from the latter in female, by the rectangular scutellar pits (circular in *formiciformis*), the long propodeal disc, which is longer than wide (wider than long in *formiciformis*), the bicolored body, which is brownish in head and mesosoma, and black in metasoma (wholly black in *formiciformis*), and the yellow legs (dark brown to black in *formiciformis*). In the forewing of the adult male and macropterous adult female, the median vein (M + Cu vein) is distinct (absent in *formiciformis*), and the pterostigma smaller than the prostigma (as large as prostigma in *formiciformis*).

**Biology.** The hosts of *Cephalonomia* species include the larvae of a wide range of Coleoptera including Curculionidae, Anthribidae, Tenebrionidae, Silvanidae, Ptinidae, Bostrichidae, Ciidae, Cucujidae, Scolytidae, Trogossitidae and Dermestidae, and Lepidoptera including Tortricidae and Autostichidae [24,37,38,39,40,41,42,43,44,45]. Some species of *Cephalonomia* are cosmopolitan and associated with stored products and are parasitoids of coleopterous larvae feeding on stored products. The host of *C. formiciformis* is beetles of the Ciidae associated with fungus [27,30], while this new species most probably attacks the larvae of Sauter anobiids, *F. sauteri*. 

**Distribution.** Taiwan (Taichung).

**Etymology.** The name is derived from the old name of Taiwan, Formosa.

## 4. Discussion

Sauter anobiids prefer to attack old books rather than modern books [14,15,46]. Kimberly et al. mentioned that books published during the period 1875–1910 may contain impermanent papers made from crude fibers, thus rendering them quite impermanent and vulnerable. This period covers the transition to different type of fibers for book papers, and some old books may have mixed good and poor papers, which may cause uneven deterioration. In this study, the damaged books we investigated are mainly dated from 1862 to 1943, a longer period than in the previous study of Kimberly et al. [46]. An additional book we examined, published in 1974, was probably infested by accident. As seen in Table 1, there is no significant difference between years; however, books published in the UK seem to have a higher damage rate than other countries, and the infestation pattern is somehow different from the books published in Japan, which is worthy of further study.

Pinniger [1] mentioned that papers with cellulose and additional materials applied to paper can provide nutrition for insects. Starch and cane sugar may be more attractive than other compounds, and cellulose itself may not be the ideal dietary component for larvae [7,10,12]. Silva et al. [10] compared different parts of book damaged by *T. herbarius* and concluded that the spine of the books was the most damaged part, especially cardboard bindings. In Moşneagu’s study in 2012 [7], *S. paniceum* favored the starch in book bindings, especially the bindings of cardboard covers. Our observations in this case are consistent with almost all the previous studies, and the materials and additives of these old books seem to influence the choice of *F. sauteri* for living and feeding. Furthermore, *F. sauteri* prefers to attack the cardboards of book hardcovers, which is similar to the previous studies of other anobiids such as *S. paniceum*, *T. herbarius* and *A. punctatum* [1,8,10].

All the books sampled in this study came from a historic library with air conditioning turned on only during the daytime or in summer. Additionally, all the books were formerly held in separate divisions of the institute before being transferred to a new integrated library. There is no information on the original conditions of these books. The high temperature and high relative humidity in Taiwan may accelerate the biodeterioration of these books and facilitate insect attack. Based on the deterioration and discoloration of these books, the original conditions might have been harsh. Books stored in a more benign environment are better preserved than those stored in poor conditions, as Kimberly et al. mentioned [46]. 

This is the first time that parasitoids of *F. sauteri* are reported. Some anobiid species are also documented as the hosts of *Cephalonomia* species, e.g., *C. gallicola* on *L. serricorne* and *S. paniceum* [29,40]. In this study, although we did not observe any living *Cephalonomia* wasps among the books or emerging from the larvae of *F. sauteri*, the high density of the wasps and Sauter anobiids co-occurring in the books and the absence of any other plausible host of the wasp places little doubt on the parasitic relationship between the two species. Bethylid wasps including *Cephalonomia* species have been reported to sting human causing painful injury or even allergic responses in many cases [21,42,47,48]. In our study, we show that *Cephalonomia* wasps are parasitoids of *F. sauteri* larvae and can occur numerously in indoor environments. It suggests that librarians or readers should take bethylid wasps into account if they are stung inside libraries. Thus, the information we offer in this article may be helpful to the identification for librarians, as well as assisting curators in the conservation of cultural heritage in the future. 

Infestation of books by *F. sauteri* created a new habitat for other arthropods. With the presence of Sauter anobiids, its parasitoids, mites, booklice and oonopid spiders, an ecological balance occurs in these damaged books.

## 5. Conclusions

Bookworms threaten the preservation of historic books. Through our investigation of old books damaged by bookworms from a historic library in Taiwan, we found that most of the damaged books were published between 1862 and 1943, and there is no significant difference in publishing years. However, a comparison of publishing countries reveals differences in bookmaking materials or methods between different countries might play an important role in infestation. For example, the old books published in the UK were found to be damaged more seriously than those of other countries and should be paid more attention during management. In addition to the bethylid wasp, the natural enemy of *F. sauteri*, the occurrence of oonopid spiders, which may utilize the tunnels caused by *F. sauteri*, is worthy of further research. 

In this study, we provide detailed ultramorphological characteristics of the larvae and adults of *F. sauteri* to facilitate the identification of this book-boring beetle. The discovery of a native parasitoid offers the chance of using this new species, *C. formosiensis*, as a potential biological control agent for *F. sauteri.* On the other hand, *Cephalonomia* species have been reported stinging human and causing painful injury in many cases, and our study points out that staff or readers in libraries, and curators who are in charge of historical archives, should consider this tiny wasp if they are stung inside libraries.

## Figures and Tables

**Table 1:** Year and country of publication of the damaged old books in this study.

Publishing Country Year	UK	Japan	USA	Germany	The Netherlands	Australia	India	Taiwan	Sum
1862				1					1
1891	1								1
1892		1							1
1893	1								1
1897	3								3
1900		1							1
1901	1	1	1						3
1902	2	1							3
1904	1								1
1905		1							1
1906		1				1			2
1907	1								1
1908			1						1
1909				1				1	2
1910				1					1
1911	1				1				2
1913	3						1		4
1914			1						1
1916	1		1		1				3
1919			1						1
1920					1				1
1922	2								2
1923	1								1
1926			1						1
1931				1					1
1934	1	2							3
1943		2							2
1974					1				1
Sum	19	10	6	4	4	1	1	1	46
